# Stromal Lymphoid Response Status in Micropapillary Urothelial Carcinomas Diagnosed in Bladder Transurethral Resections and its Comparison with Conventional Urothelial Carcinomas

**DOI:** 10.5146/tjpath.2020.01497

**Published:** 2021-01-15

**Authors:** Ezgi Hacıhasanoğlu, Uğur Yücetaş, Oğuzhan Okçu, Kemal Behzatoğlu

**Affiliations:** Department of Pathology, Yeditepe University Faculty of Medicine, Istanbul, Turkey; Department of Urology, University of Health Sciences, Istanbul SUAM, Istanbul, Turkey; Department of Pathology, Recep Tayyip Erdogan University, Faculty of Medicine, Rize, Turkey; Acibadem Health Group, Istanbul, Turkey

**Keywords:** Micropapillary urothelial carcinoma, Stromal lymphoid response, Transurethral resection, Urothelial carcinoma, Bladder

## Abstract

***Objective:*** Micropapillary urothelial carcinoma is an aggressive variant of urothelial carcinoma. Evidence suggests that the relationship between the tumor and inflammatory cells is important in tumor progression and the treatment response. We evaluated the stromal lymphoid response in micropapillary urothelial carcinomas and compared it with conventional urothelial carcinomas.

***Material and Method:*** Among bladder transurethral resection materials diagnosed as ‘invasive urothelial carcinoma’ between January 2010-March 2017, cases with at least 5% micropapillary urothelial carcinoma were evaluated for age, gender, grade, stage, micropapillary urothelial carcinoma percentage, presence/percentage of accompanying conventional urothelial carcinoma/urothelial carcinoma variants, in situ urothelial carcinoma/micropapillary urothelial carcinoma, lymphovascular invasion, necrosis, and stromal lymphoid response. Stromal lymphoid response was scored as 0-1-2-3. All parameters were evaluated in 50 pure conventional urothelial carcinomas.

***Results:*** Among 47 micropapillary urothelial carcinomas, 41 were male. The mean age was 69 years. pT1/pT2 was 23/24. Six cases were pure MPUC. Lymphovascular invasion was present in 8, necrosis in 9 cases. Stromal lymphoid response was present and scored as 1-2-3 in 32 micropapillary urothelial carcinomas (68.1%) and 48 conventional urothelial carcinomas (96%). Micropapillary urothelial carcinomas had significantly higher lymphovascular invasion and pT2 rates and lower stromal lymphoid response.

***Conclusion:*** Low stromal lymphoid response in micropapillary urothelial carcinomas can be responsible for the poor clinical outcome and impaired response to treatment of these tumors. This is the first study in the English literature to demonstrate a lower stromal lymphoid response rate in micropapillary urothelial carcinomas compared to conventional urothelial carcinomas.

## INTRODUCTION

Micropapillary urothelial carcinoma (MPUC) is an aggressive variant of urothelial carcinoma (UC) described by Amin et al. in 1994 (1,2). It is a rare variant with reported incidence being 0.6-6% among UCs (1–4). MPUC is more frequently encountered in males and in the 6th and 7th decade (1,2,5). MPUC is usually diagnosed at an advanced stage (1). Microscopically the tumor is characterized by tightly packed cell clusters without fibrovascular cores and surrounding lacuna which resemble small dilated lymphatic channels (2,6,7). Lymphovascular invasion is an early finding in MPUCs and thus metastasis is more frequently encountered (2,3,7–9).

There is increasing evidence that the relationship between tumor cells and inflammatory cells has great importance in the development and progression of tumors (10,11). The lymphoid response to the tumor has also been shown to have an impact on the treatment response and survival in various cancer types, including UC (10,12–15). The status of the lymphoid response in the tumor stroma can be an important factor responsible for poor clinical outcome and impaired treatment response in MPUCs.

Our aim in this study was to document the stromal lymphoid response and other histopathological features of MPUCs diagnosed in bladder transurethral resection (B-TUR) materials, and to compare these parameters with conventional UCs.

## MATERIAL and METHODS

The electronic database of a single center pathology department was scanned for cases diagnosed as ‘Invasive urothelial carcinoma’ in B-TUR materials between January 2010-March 2017. All hematoxylin-eosin (H&E) stained slides were retrieved from the archive and reviewed for MPUC. There is no specified criterion for the cutoff proportion of the micropapillary component to qualify as MPUC, and 5% or 10% has been suggested as the lower limit (16). Most series in the literature have included cases with <10% micropapillary component and it is reported that any amount of micropapillary component is associated with a poor outcome (4,9,17). In the light of this information, cases with a minimum 5% micropapillary component were included in the study. For patients with a MPUC diagnosis in recurrent B-TURs, only the first B-TUR materials with MPUC were included in the study. Demographic data of the cases were obtained from the hospital electronic database.

Histological grading and staging were done according to World Health Organization (WHO) 2016 Classification of Urogenital Tumors (1). All cases were evaluated in terms of age, sex, histological grade, stage, MPUC percent, accompanying conventional UC and other UC variants, in situ UC, in situ MPUC, lymphovascular invasion, and necrosis. Lymphoid response in the tumor stroma was assessed in H&E stained slides, with a methodology similar to that used in the study by Klintrup et al. in colorectal carcinomas (18). The tumor stroma was evaluated with the x10 objective, and a four-degree scale of 0-1-2-3 was used for scoring. A score of 0 was given when there were no/hardly any mononuclear inflammatory cells identified. A score of 1 was given when mild and patchy infiltration of mononuclear inflammatory cells were spotted. Score 2 was given when there was widespread mononuclear inflammatory cell infiltration but the stromal fibrous tissue was also recognizable in the background. Score 3 denoted very extensive mononuclear inflammatory cell infiltration so that the background fibrous tissue could not be distinguished. The inflammatory response score was independently assessed by three pathologists. When two or all the pathologists had the same score, that score was accepted as the final score. If all three pathologists had different scores, the final score was given after a consensus evaluation. For the facilitation of the statistical analysis, the four-degree scale was reduced to a two-degree scale: score 0 was accepted as negative for stromal lymphoid response, and scores 1, 2 and 3 were combined and regarded as positive for stromal lymphoid response.

All these parameters were also evaluated in 50 cases of randomly selected invasive conventional urothelial carcinoma cases diagnosed in B-TUR materials between January 2010-March 2017.

IBM SPSS Statistics for Windows, version ٢٢.٠ (IBM Corp., Armonk, N.Y., USA), was used in the statistical analysis. The Kolmogorov-Smirnov test was used to assess whether a variable followed a normal distribution or not. The independent samples t-test and the Mann-Whitney U test were used in the analysis of quantitative independent data. The chi-square test was used in the analysis of qualitative independent data. P values of less than 0.05 were regarded as statistically significant.

## RESULTS

Among 1440 B-TUR materials with an ‘invasive urothelial carcinoma’ diagnosis between January 2010- March 2017, 59 cases (4.1%) had more than 5% MPUC component. Twelve patients had more than one B-TUR material with MPUC component. Only the first B-TUR materials for these patients were taken into consideration and the following biopsies were excluded. Thus, a total of 47 MPUC cases were included in the study. Distributions of the MPUC cases and conventional UC cases were not significantly different (p>0.05). Demographic and histopathological features of the MPUC and conventional UC cases and statistical comparison of the findings between the two groups are summarized in Table 1.

**Table 1:** Comparison of demographic and histopathological findings between conventional UC and MPUC cases.

		**Conventional UC**		**MPUC**		**p**	
** **		**Ave.±s.d.**	**Median**	**Ave.±s.d.**	**Median**		
Age		66.9 ± 9.7	66.0	69.0 ± 10.4	69.0	0.31	t
		**n**	**%**	**n**	**%**		
Sex	Female	7	14.0	6	12.8	0.858	X²
	Male	43	86.0	41	87.2		
Stage	pT1	45	90.0	23	48.9	**<0.001**	X²
	pT2	5	10.0	24	51.1		
In situ UC	Negative	20	40.0	15	31.9	0.407	X²
	Positive	30	60.0	32	68.1		
ALI	Negative	50	100.0	39	83.0	**0.002**	X²
	Positive	0	0.0	8	17.0		
Necrosis	Negative	42	84.0	38	80.9	0.684	X²
	Positive	8	16.0	9	19.1		
SLR	Negative	2	4.0	15	31.9	**<0.001**	X²
	Positive	48	96.0	32	68.1		

**UC:** Urothelial carcinoma, **MPUC:** Micropapillary urothelial carcinoma, **ALI:** Angiolymphatic invasion, **SLR:** Stromal lymphoid response, t: t-test, X2: Chi-square test.

### Demographic and Histopathological Findings in MPUC Cases

Forty-one cases were male and 6 were female. The age range was 43-89, with a mean age of 69 and a median of 69 years. Twenty-three cases were stage pT1 and 24 cases were pT2. All cases had high grade histological features. The percentage of MPUC component in the cases were between 5-100%, with a mean of 37.8%. Six cases had pure micropapillary morphology. In 41 cases, MPUC was accompanied by conventional UC. One or more UC variant other than MPUC was present in 13 cases (27.6%). The most common UC variant accompanying MPUC was the nested variant (6 cases). Other accompanying variants were as follows: poorly differentiated variant (3 cases), sarcomatoid variant (1 case), lipid-rich variant (1 case), and pseudoangiomatous variant (1 case). Areas of glandular differentiation were seen in 7 cases. Squamous and trophoblastic differentiation was present in 2 cases, each. In situ UC was present in 32 cases and in situ MPUC in 2 cases. Lymphovascular invasion was detected in 8 cases. Necrosis was seen in 9 cases.

**Figure 1:**
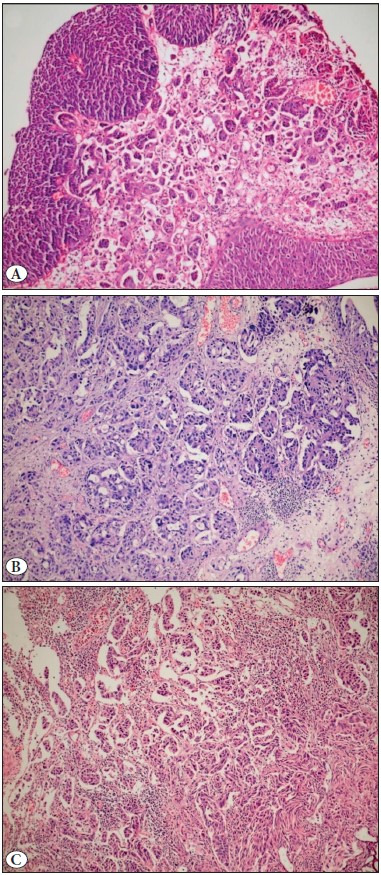
Stromal lymphoid response scoring. **A)** Score 1 lymphoid response was defined as mild and patchy infiltration of mononuclear cells (H&E; x10). **B)** Score 2 lymphoid response was defined as widespread mononuclear inflammatory cell infiltration with the stromal fibrous tissue still recognizable in the background (H&E; x10). **C)** Score 3 lymphoid response was defined as very extensive inflammatory infiltration so that the background fibrous tissue could not be distinguished (H&E; x10).

Evaluation of the stromal lymphoid response was done independently by three pathologists. In 36 cases, all three pathologists had the same score. In 7 cases, two pathologists had the same opinion and that was decided as the final score. In 4 cases, a consensus meeting was held for the final score. In all four of these cases, the given scores by the pathologists before the consensus meeting were 0, 1, and 2. The common property of these cases was the heterogeneity of stromal lymphoid infiltration. After the consensus meeting, 2 cases were assessed as score 1 and 2 cases were assessed as score 2. As a result, and according to the four-degree scale evaluation of stromal lymphoid response as 0-1-2-3, 15 cases did not show a lymphoid response (score 0) whereas 18 cases had a score of 1, 9 cases had a score of 2 and 5 cases had a score of 3 for the lymphoid response. Among the 6 pure MPUC cases, the stromal lymphoid response score was 0 in 4 cases and 1 in 2 cases (Figure 1).

### Demographic and Histopathological Findings in Conventional UC Cases

In 50 conventional UC cases, the age range was 48-89 years, with a mean of 66.9 and median of 66 years. Forty-three patients were male and 7 were female. All cases were invasive UC cases with high grade nuclear features. Forty-five and 5 cases were pT1 and pT2, respectively. In situ UC was present in 30 of the control cases (60%). Necrosis was detected in 8 cases, whereas none of the cases had lymphovascular invasion.

Evaluation of the stromal lymphoid response was done independently by three pathologists. All three pathologists had the same score in 33 cases. In 12 cases, two pathologists had the same opinion and that was decided as the final score. In 5 cases, the final score required a consensus meeting. The given scores by the pathologists in these cases were 0, 1 and 2. Similar to what was encountered in the MPUC cases, the common property of these cases was the heterogeneity of stromal lymphoid infiltration. Three cases were given a score of 1 and 2 cases were given a score of 2 after the consensus meeting. Consequently, stromal lymphoid response was scored as 0 in 2 cases, 1 in 33 cases, 2 in 11 cases and 3 in 4 cases.

### Statistical Analysis of the Findings Between Two Groups of Cases

Statistical analysis showed no significant difference in terms of patient age and sex between MPUC cases and control cases. Also, no statistically significant difference was seen between the two groups in terms of in situ UC and necrosis.

The ratio of pT2 was significantly higher in the MPUC group (p<0.001). The lymphovascular invasion rate of MPUC cases was also significantly higher than in the control group (p<0.05). In order to make the scoring system more reproducible and to facilitate the analysis, the four-degree scale was converted to a two-degree scale. Score 0 was accepted as negative for stromal lymphoid response, and scores 1, 2, and 3 were combined and regarded as positive for a stromal lymphoid response. Stromal lymphoid response presence was significantly lower in the MPUC group than the control group (p<0.001).

## DISCUSSION

MPUC is a rare histological variant of UC with aggressive clinical outcome and poor prognosis (1,19). In our series, 4.1% of all UC cases had a micropapillary component, consistent with the reported incidence of 0.6-8% among UCs (1–4). The age range was 43-89, with a mean age of 69, similar to the literature (1,2,5). MPUC is known to show a male predominance (1,2,7). The male/female ratio was 6.8:1 in our study. MPUC usually presents with high stage disease and shows high grade histology (1–3,19). In this study, 23 cases were stage pT1 and 24 cases were pT2. All cases had high grade histological features. Lymphovascular invasion is a frequent finding in MPUCs; very high lymphovascular invasion rates were reported by Amin et al., Alvarado-Cabrero et al. and Johansson et al., respectively, 100%, 89% and 75% (2,3,7). The lymphovascular invasion rate was 17% in our study, relatively low compared to the literature, but it was significantly higher than the conventional UC cases.

It has been showed in many studies that the immune system can provide a defense system against cancer in tumorigenesis process, but it may also facilitate cancer development (11,20,21). As in many other types of malignancies, chronic inflammation takes place in the pathogenesis of the urothelial carcinoma and it has a double-sided role (22). On one side, chronic inflammation is a well-known risk factor for bladder carcinoma development. The best example for this can be *Schistosoma haematobium* infection in bladder carcinogenesis (1). On the other hand, inflammation is induced by intravesical Bacillus Calmette-Guerin therapy in bladder cancer treatment and cancer recurrence is prevented (22).

An inflammatory response is developed against UC, as with many other cancers in the human body (10). Several studies report that some cancers which have an inflammatory response developed against them have better outcomes and are associated with longer patient survival (18,23–25). In a recent study by Liu et al., higher numbers of tumor infiltrating lymphocytes was found to be related with longer survival in bladder urothelial carcinoma (26). Lymphocyte infiltration in the tumor has also shown to be associated with the chemotherapy response (27). The mechanisms behind the association between lymphoid response and survival and the therapy response have not been fully elucidated yet.

In our study, the rate of stromal lymphoid response was 96% in conventional UCs and 68.1% in MPUCs, and the difference between the two groups was statistically significant. This finding can be explained by several probable reasons. One of them can be the rapid progression of MPUC so as not to allow development of lymphoid response in the tumor. Another reason can be the possible secretion of special chemical or immune mediators from MPUC in order to inhibit the lymphoid response in the stroma. Lower lymphoid response in MPUCs can also be related to their lower response to standard UC therapy. Regardless of the mechanism behind this, these tumors are more aggressive than conventional UCs and their survival rates are lower.

To the best of our knowledge, there are no studies in the English literature investigating the relationship between MPUC and stromal lymphoid response. This is the first study to demonstrate a lower stromal lymphoid response rate in MPUCs compared to conventional UCs. The findings of our study need to be supported or opposed by other studies. This area requires further research with more cases.

## CONFLICT of INTEREST

The authors declare no conflict of interest**.**

## FUNDING

The authors received no financial support for the research, authorship, and/or publication of this article*.*
